# Does the Urban Agglomeration Policy Reduce Energy Intensity? Evidence from China

**DOI:** 10.3390/ijerph192214764

**Published:** 2022-11-10

**Authors:** Rui Ding, Tao Zhou, Jian Yin, Yilin Zhang, Siwei Shen, Jun Fu, Linyu Du, Yiming Du, Shihui Chen

**Affiliations:** 1 College of Big Data Application and Economics (Guiyang College of Big Data Finance), Guizhou University of Finance and Economics, Guiyang 550025, China; 2 Guizhou Key Laboratory of Big Data Statistical Analysis, Guizhou University of Finance and Economics, Guiyang 550025, China; 3 Key Laboratory of Green Fintech, Guizhou University of Finance and Economics, Guiyang 550025, China; 4 West China Modernization Research Center, Guizhou University of Finance and Economics, Guiyang 550025, China; 5 School of Water Conservancy and Civil Engineering, Northeast Agricultural University, Harbin 150050, China

**Keywords:** the urban agglomeration policy, energy intensity, Difference-In-Differences model, the mediation mechanism

## Abstract

With the expansion of the scale of China’s economy and the acceleration of urbanization, energy consumption is increasing, and environmental degradation and other problems have arisen. In order to solve such prominent problems, China proposed the “carbon peak” and “carbon neutral” targets in 2020. Although there are research conclusions about the impact of urbanization on energy intensity (*EI*), conclusions about the impact of the urban agglomeration policy (*UAP*) on *EI* are still unclear. Therefore, the article studies the impact of the urban agglomeration policy on *EI* in 279 prefecture-level cities by constructing a Difference-In-Differences (DID) model and mediating effect model. The results show that *UAP* has a significant effect on reducing *EI*, but their effects are different with the impact of urban heterogeneity, and the urban agglomeration policy of “Core” cities is less effective than those of “Edge” cities. From the perspective of the influencing mechanism, *UAP* takes green innovation capability as the intermediary variable to influence *EI*. The placebo test, PSM-DID regression, counterfactual test, and instrumental variable method all reflect the robustness of the research conclusions. Based on this, the paper puts forward some suggestions for urban agglomeration planning and green technology innovation.

## 1. Introduction

Energy has made an indelible contribution to the global economy in the early stage of economic development. According to the forecast data of the U.S. Energy Information Administration, global energy consumption is expected to increase by nearly 50% from 2018 to 2050 (https://www.eia.gov/, accessed on 8 April 2022). In addition, on the basis of the data released by the International Energy Agency, carbon dioxide from oil, gas, and coal consumption increased by about 44.34 percent between 2000 and 2018 (https://www.iea.org/data-and-statistics/data-tools/energy-statistics-data-browser?country=WORLD&fuel=CO2%20emissions&indicator=CO2BySource/, accessed on 13 April 2022). This increasing energy consumption has brought about global warming, biodiversity reduction, water pollution, and many other environmental problems. Although the proportion of clean energy use is increasing, traditional fossil fuels are still dominant. Even by 2040, fossil fuels will account for more than two-thirds of global primary energy (https://www.worldenergy.org/, accessed on 13 April 2022). Therefore, in order to effectively cope with the environmental problems caused by energy consumption, countries around the world are actively exploring various ways to improve energy efficiency and creating a healthy energy environment for the realization of sustainable development goals.

With the acceleration of urbanization, cities have become the core of economic development [1]. Meanwhile, with the rapid growth of the urban population, urban residents have become the main force of fossil energy consumption, so the development of urbanization will have a significant impact on energy consumption [2,3,4]. By gathering surrounding resources and elements, cities continue to expand their scales, which makes energy elements flow to cities, resulting in China’s urban energy consumption has exceeded 80% of the country. On the other hand, cities also face the problems of energy consumption surge, pollutant emission increase, and environmental deterioration. Urban development increases energy consumption; when several cities gradually develop as an urban agglomeration, the cost of information exchange within the urban agglomeration will be reduced, and the agglomeration of talents and scientific and technological factors will be accelerated, which will contribute to the improvement of innovation ability and alleviate energy consumption. Urban agglomeration development realizes economic development of urban agglomeration through the integration of various resources, and economic activities of urban agglomeration are important factors affecting energy efficiency [5,6]. It can be seen that the development of urban agglomeration is bound to have the contradiction of increasing and reducing energy consumption. As the official planning for the development of urban agglomeration, urban policy not only includes the direction of social and economic development but also puts forward the requirements of energy utilization and environmental protection. In pursuit of coordinated economic, social, and environmental development, the number of urban agglomerations in China has seen a spurt of growth, with the number of state-level planning approvals increasing from one to nine between 2015 and 2018. However, it remains to be seen whether this policy will help solve the problem of energy consumption.

**Figure 1:**
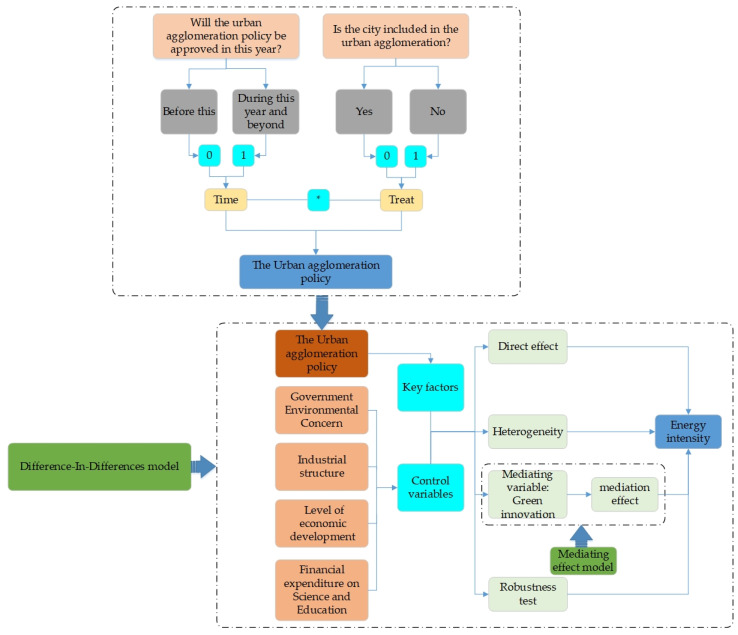
Research framework diagram.

At present, most studies on urban agglomeration focus on the coordinated development and economic growth of urban agglomeration, and few focus on the impact of *UAP* promulgation on energy factors. Considering these facts, the paper puts forward the following three questions. The first question is whether urban agglomeration policy can reduce *EI*? The second question is, does the urban agglomeration policy reduce *EI* differently depending on urban positioning? The third question is, what is the main mechanism through which *UAP* can reduce *EI*?? Through the study of the above problems, we can have a more comprehensive and profound understanding of the new direction of energy development in urban agglomeration. It also provides a theoretical and practical basis for the study of the energy development mechanism of urban agglomeration. This article is mainly from the following aspects of research. Firstly, the specific situation of the research object is explained, highlighting the importance of the article research. Secondly, to sort out the previous research, design the research hypothesis of the article. Thirdly, construct the DID model and select relevant index data. Fourthly, benchmark regression was conducted to analyze the heterogeneity effect and mediating effect, explore the mechanism of action and compare and discuss the relevant conclusions. Fifthly, draw relevant conclusions and suggestions. The specific research design is shown in Figure 1.

## 2. Theoretical Mechanism Analysis

Although Lewis repeatedly acknowledged the relevance of the natural resource base as an important factor in the transition from a capitalist environment to modern times, he did not explicitly state the situation of countries with mineral resource wealth in his model of the dual economy theory [7,8]. In Lewis model of dual economy theory, he explained that resources such as labor eventually flow to the modern industrial sector. At this time, the proportion of the agricultural sector will decline, and the proportion of the industrial sector will increase. His research, however, assumes “an unlimited supply of energy, labour and other resources for the expansion of the industrial sector”. Nowadays, the industrial sector has been faced with the problems such as the shortage of energy resources in the process of development and can no longer effectively meet the unlimited supply of energy and resources. Sadik-Zada [9] proposed an improved Lewis model, in which the elements of natural resources, trade, and institutions neglected in the original Lewis model are included, and the approximate determinants of economic growth are also considered. As a kind of resource element, energy plays an indispensable role in the process of economic growth. From the perspective of regional development in ancient China, administrative divisions that ignore the elements of geographical units cut off regional connections and economic growth [10], and the demand for energy factors in various regions is low. Until the founding of the People’s Republic of China, China’s regional development mode has a qualitative leap. The mode of promoting the urbanization process from small towns to “urban cluster” development mode, and this urban cluster development has become an important form of urban economic development in China. The development of urban agglomeration requires a large number of energy factors, leading to the flow of energy factors into the city, and urban energy consumption keeps rising. While promoting regional economic development, urban agglomeration increases energy consumption, but its impact on energy intensity is still unknown. Therefore, it is urgent to study the impact of *UAP* on *EI* from the state-level urban agglomerations that have been approved in China.

As for the influencing factors of *EI*, scholars have studied from the perspectives of economic growth, industrial structure, technological progress, trade openness, and financial developments were studied [11,12,13,14,15].

From the historical development of China, there are serious market segmentation and local protectionism in China [16]. Economic competition between most local governments intensifies resource competition and market fragmentation [17]. Competition mechanisms for realizing economic development goals made regional *EI* surge. With the improvement of the urbanization level and wide-area marketization level, urban agglomeration development has entered the stage of effective allocation of resources by integrating regional resource elements [18]. This not only improves the efficiency of resource utilization but also forms the agglomeration economic development model [19]; this model has a significant impact on regional *EI* [20]. First of all, urban agglomeration development breaks the original economic development thinking of administrative divisions and alleviates resource competition. Secondly, the economic development of each city has changed from extensive to intensive mode. In order to achieve this intensive development goal, the upgrading of industrial structure and the promotion of regional innovation ability have reduced *EI* to a certain extent. Considering the role of urbanization level in the process of urban agglomeration, some scholars have conducted relevant studies on urbanization and *EI*. Such as Bilgili [21] found that the level of urbanization reduced *EI* in Asian countries, but Sadorsky [22] believed that urbanization had a dual impact on *EI*; urbanization and industrialization would collectively reduce *EI* as income growth offsets them. Existing studies have shown that urban agglomeration development and urbanization have a significant impact on *EI*, and *UAP*, as the government’s official planning for urban agglomeration development, should also have more or less impact on *EI*. Just as Kong et al. [23] studied the impact of state-level urban agglomeration policies on economic growth and environmental pollution, they found that *UAP* effectively promoted economic growth and mitigated environmental pollution. Based on existing studies, the article puts forward the following **hypothesis 1**.

**Hypothesis 1** **(H1).** *UAP implementation helps reduce EI*.

The implementation of *UAP* is of great significance in reducing *EI*; if we generalize that *UAP* have the same impact on *EI* of all types of cities, it will ignore the heterogeneity of the development of each city and fail to give full play to the policy effectiveness. Most countries do not “fight alone” for the implementation of a certain policy but “go together” with other policies. Therefore, based on this idea, the article considers the national positioning of regional core cities when considering *UAP*. Yu et al. [24], considering the influence of different regions and periods, divided the region into the eastern, central, western and northeastern regions to explore the different influencing factors of energy efficiency and found that state intervention has the greatest impact on the northeastern region, while the industrial structure has a significant impact on the central region. Hang et al. [25] divided cities into five groups of high and low energy efficiency based on the analysis of the structure of the rich and the poor of the World Bank to study the reasons for the difference in energy efficiency, and found that cities in the middle-income group were mainly dominated by enterprises with high energy consumption and high pollution, which led to their low energy efficiency. This kind of heterogeneity analysis based on geographical location and classification methods published by authoritative institutions reflects, to some extent, the necessity for scholars to study urban heterogeneity [26]. Based on this, the article puts forward **hypothesis 2**.

**Hypothesis 2** **(H2).** *The effect of UAP implementation on reducing EI is affected by urban heterogeneity.*

*UAP* can reduce *EI*, but the mechanism is still unknown. From previous studies, some scholars have studied the path of affecting *EI*, and most of their studies show that technological progress is the key mediating factor affecting *EI*. For example, Pan et al. [14] analyzed the influencing factors of *EI* in Bangladesh by constructing a path model and found that industrialization indirectly reduced *EI* through technological innovation. Ma et al. [27] used LMDI technology to decompose China’s *EI* during the research period and found that technological progress led to the decline of *EI*. Voigt et al. [28] believed that the decline of global *EI* was mainly due to technological progress, which was a key factor in improving energy efficiency. Sun et al. [29] found that the development of energy technology plays a crucial role in improving energy efficiency. Ouyang et al. [30] see energy technology innovation as an important way to improve energy efficiency. Wang [31] believes that urban agglomeration integration will indirectly affect urban pollution emissions through energy consumption and green technology innovation. According to the above research, the intermediate factor affecting energy intensity is mainly technological progress, and technological progress can be mainly divided into clean technological progress and non-clean technological progress [32], and clean technological progress is more important for energy conservation and emission reduction. Based on this, the paper puts forward **hypothesis 3**.

**Hypothesis 3** **(H3).** *UAP implementation indirectly affects EI through green innovation*.

## 3. Study Design

As a general strategy of regional coordinated development, *UAP* has an important impact on the urban economy, society, and environment. Studies on the impact of *UAP* on *EI* are scarce at present. Therefore, this article studies the impact of *UAP* on *EI* and explore the net effect of *UAP* on *EI*. In addition, it analyzes whether the impact effect of *UAP* on *EI* is consistent under urban heterogeneity. Finally, the paper studies the impact path of *UAP* on *EI*. Through the above research and analysis, the effects of *UAP* on *EI* are obtained, which is of great significance to urban agglomeration planning and energy economic development.

### 3.1. Model Building

In order to study the impact of *UAP* on *EI*, *UAP* is regarded as a quasi-natural experiment. *UAP* represents an action direction for the common development of regional cities. Therefore, *UAP* is regarded as a new policy intervention, and the effect of *UAP* on reducing *EI* is evaluated by measuring the change in *EI* before and after the implementation of experimental group and control group policies. As a differential model commonly used in policy effect assessment, it can effectively measure the average treatment effect of the treatment group before and after policy implementation under the premise of meeting the assumption of a parallel trend. Based on this, the paper constructed a DID model:

(1) $$\begin{array}{c} EI_{i,t}=\alpha_{1}+\beta_{1}UAP_{i,t}+\sum_{m=1}^{4}\theta_{m}control_{n}+\mu_{i}+\lambda_{t}+\varepsilon_{i,t} \end{array}$$

Among them, *EI* is the logarithmic form of energy consumption intensity, $UAP_{i,t}$ is the dummy variable of urban agglomeration policy, $UAP_{i,t}=Treat_{i,t}\times Time_{i,t}$, $control_{n}$ is the control variable of government environmental protection attention, industrial structure, economic development level and per capita expenditure on science and education, $\mu_{i}$, $\lambda_{t}$ represent city fixed effect and time fixed effect, $\varepsilon_{i,t}$ are random disturbance terms. $\alpha_{1}$ is a constant term, $\beta_{1}$, $\theta_{m}$ are regression coefficients of variables, and *i* and *t* represent city and time. $\beta_{1}$ is the regression coefficient that is mainly concerned in this paper. If $\beta_{1}$ is significantly negative, it indicates that the *UAP* can reduce the *EI* of the policy-imposed city.

### 3.2. Variables and Data Sources

#### 3.2.1. Dependent Variable (*EI*)

Energy intensity (*EI*) represents the input–output efficiency of energy. Based on the research method of Liao [33], *EI* is expressed as the natural logarithm of the ratio of total energy consumption to GDP, i.e., *EI* per CNY 100 million of GDP. In this paper, the total amount of coal consumption is expressed by multiplying the total amount of natural gas, liquefied petroleum gas, and electricity consumption by the conversion coefficient of standard coal per ton.

(2) $$EI_{i,t}=\frac{E_{i,t}}{GDP_{i,t}}$$

where *EI* represents energy intensity, *E* represents total energy consumption, and GDP represents the economic development level of prefecture-level cities.

#### 3.2.2. Independent Variable (*UAP*)

**Figure 2:**
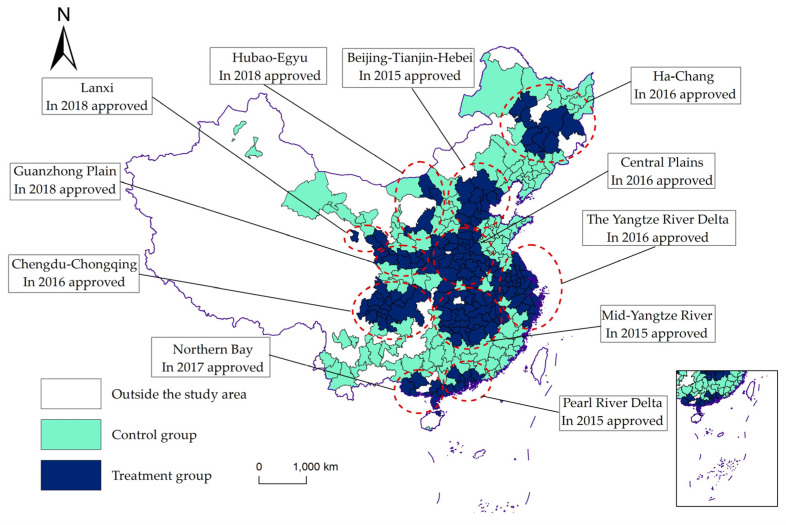
Approval time of urban agglomerations and the study area.

Urban agglomeration policy (*UAP*) represents a policy preference for urban cluster development in China and is a dummy variable of 0 and 1.

(3) $$UAP_{i,t}=Treat_{i,t}\times Time_{i,t}$$

where $Treat_{i,t}$ are the dummy variables of the treatment group. If city *i* is within the urban agglomeration planning scope, $Treat_{i,t}=1$, and $Treat_{i,t}=0$ for outside the scope. $Time_{i,t}$ are time dummy variables. If city *i* is included in urban agglomeration planning in the *t* year, then $Time_{i,t}=1$ in the *t* year and after; otherwise, it is 0. *UAP* is based on the official reply of the State Council and the information document issued by the National Development and Reform Commission. Considering that the Beijing-Tianjin-Hebei urban agglomeration has not been officially approved by the State Council, the paper takes the Outline of the Beijing-Tianjin-Hebei Coordinated Development Plan as the benchmark. The Guangdong-Hong Kong-Macao Greater Bay Area will be adjusted to 2015 based on the approval date of the Pearl River Delta City Cluster. The specific approval time and range of each urban agglomeration are shown in Figure 2.

#### 3.2.3. Control Variables

*UAP* was taken as the core explanatory variable to study its effect on *EI*, and the influences of government, economy, and society should be fully considered. Therefore, the paper selected the government’s environmental protection attention, industrial structure, urban economic development level, and scientific and educational financial expenditure as relevant control variables.

(1) Government Environmental Concern (GEC). The environmental protection concern of the government is the environmental regulations adopted to save energy, reduce emissions and protect the environment [34]. To some extent, the government’s concern about environmental protection will make the economy develop towards the direction of low energy consumption and may improve energy use efficiency. Farooq [35] measured the government’s green environmental concerns with four indicators, including carbon tax rates and renewable energy generation. Based on the data availability reference, Chen et al. [36] counted the word frequency of “environmental protection” in the reports of prefecture-level city governments and calculated the natural logarithm of its word proportion in the full text of government work reports to measure the government’s attention to environmental protection.

(2) Industrial structure (IS). Although China’s industrialization and urbanization make the secondary industry become the leading force of national economic development, the energy consumption of the secondary industry also occupies the vast majority of the total energy [13]. Industrial structure has a direct impact on *EI* because industrial development has a great demand for energy consumption. If the industrial structure is dominated by secondary industry, energy consumption is greater. Therefore, this paper refers to the research method of Pan et al. [37] and takes the proportion of the GDP of the secondary industry in the total GDP as the expression of industrial structure. A larger value indicates that the economic development structure is dominated by the industrial structure, and energy consumption may be higher.

(3) Level of economic development (PGDP). The conventional view is that *EI* declines with economic growth, with faster economic growth contributing to lower *EI* [38]. Emir [39] used the real GDP index to measure the economic growth of Romania, and Chen (2019) used the GDP growth rate as the indicator to measure the level of economic development [40]. Filipović et al. [41] and Jimenez and Mercado [42] believe that per capita GDP, energy price, and energy tax are important decisive factors of *EI*. At the same time, considering the analysis bias caused by the influence of regional population, the paper takes the logarithm form of per capita GDP as the measurement index of economic development level.

(4) Financial expenditure on Science and Education (FESE). Spending on science and technology will increase spending on research and development, which in turn will help mitigate environmental pollution [43]. Considering that *EI* is closely related to science, technology, and talents, this paper chooses the expenditure on science education as a control variable to study *EI*. Mainly, the expenditure on science education represents the government’s resource inclination, including R&D expenditure and talent training. The more R&D expenditure and scientific and technological talents, the less energy consumption for economic and social development. Referring to the indicator measurement method of Bu [44], the elimination of population impact is expressed in the logarithmic form of per capita expenditure on science and education.

#### 3.2.4. Mediating Variable (GIC)

Green Innovation Capability (GIC). The development of urban agglomeration is the agglomeration of various resources, which will greatly promote the development of science and technology. The development of science and technology will improve the quality of economic development and change the problem of energy consumption in the process of economic development. The main methods to measure green innovation ability include DEA measurement of green innovation efficiency and the number of green invention patents directly adopted [45,46]. As the number of green invention patent applications has a high technical threshold, it is required to promote the research and development, promotion, and application of green technology at the same time as green product innovation, which reflects the ability of green innovation at a higher level [47,48]. Therefore, referring to the indicator measurement method of Wurlod and Noailly [48], this paper selects the number of green invention patent applications as the indicator of urban green innovation ability and takes the natural logarithm of it.

The variable data above came from the statistical yearbooks of prefectural cities, the work reports of municipal governments, and the EPS data platform. Among them, the number of green invention patent applications came from the national intellectual property database, which was matched by the Green List of international Patent Classification of The World Intellectual Property Organization (WIPO).

## 4. Analysis of the Empirical Results

### 4.1. Descriptive Statistical Analysis

In order to determine whether outliers exist, descriptive analysis is conducted for each variable, and the descriptive results are shown in Table 1.

Table 1 shows the descriptive statistical results of each variable. It can be seen from the table that the logarithmic mean value of *EI* is −2.882, and the standard deviation is small, indicating that the *EI* values of each region have small differences in each year. The core explanatory variable *UAP* is the dummy variable of urban agglomeration policy, and its mean value is close to 0, indicating that Urban agglomeration planning in China is still concentrated in a small part of regions. Except for the industrial structure, the difference between the mean value and the maximum value of the control variables is small, indicating that there is no obvious outlier value of the variables, and there is a large development gap between the industrial structure.

### 4.2. Overall Effect Analysis

#### 4.2.1. Parallel Trend Test

**Figure 3:**
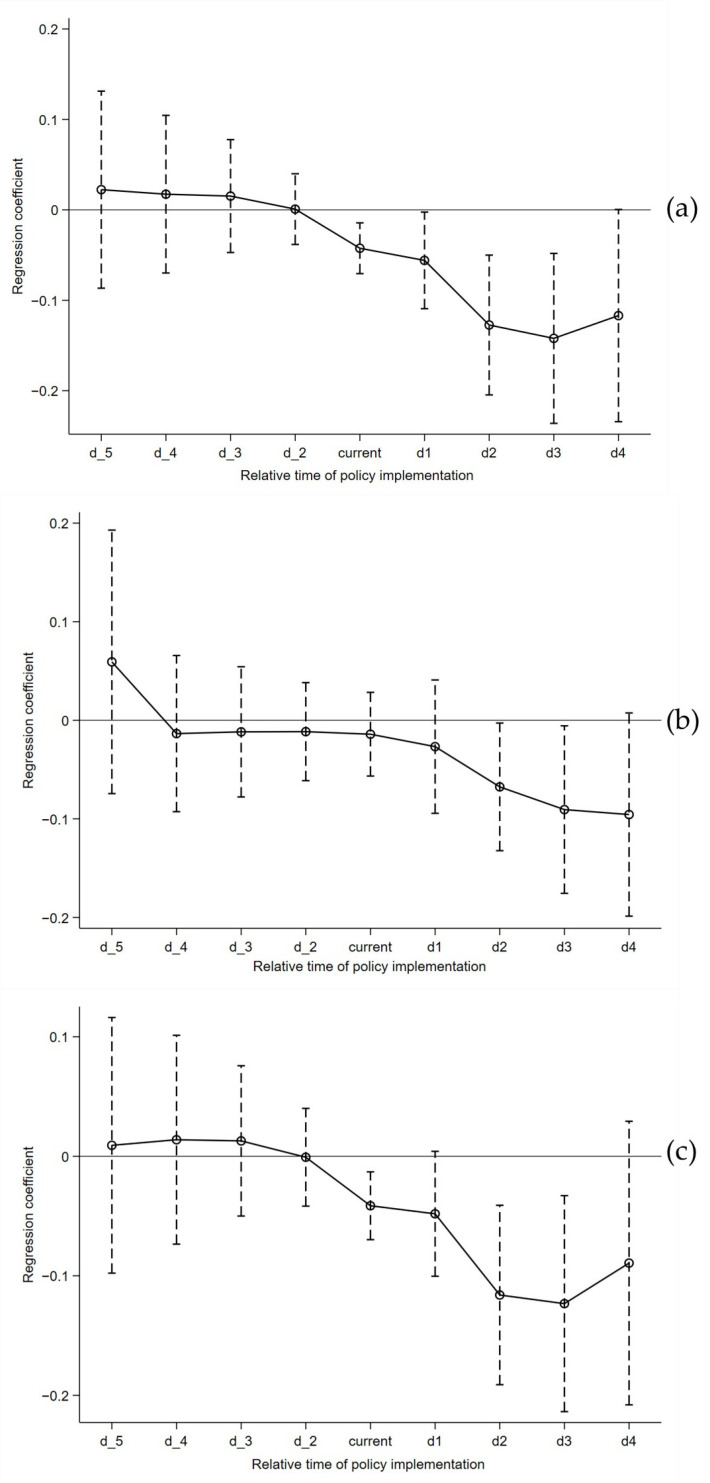
The parallel trend test results are as follows: d_2–d_5 represents the second–fifth period before the implementation of the policy, and d1–d4 is the first–fourth period after the implementation of the policy. (**a**) indicates the parallel trend test result of the whole sample cities, (**b**) is the parallel trend test result of the “Core” cities, and (**c**) means the parallel trend test result of the “Edge” cities.

In order to reduce the error of regression results, the parallel trend test is carried out before the DID model regression, which is also an important prerequisite of the DID model. Determine whether there is a significant difference in *EI* between the cities in the treatment group and the cities in the control group. If there is no difference, the parallel trend hypothesis test is satisfied. Beck et al. [49] took the current period of policy implementation as the base period for the parallel trend test. Due to the geographical particularity of urban development, the development trend of urban agglomeration may already exist before the announcement of *UAP*. Therefore, the paper takes the period before the policy implementation as the base period to conduct a parallel trend test, and the test results are shown in Figure 3. It can be seen from Figure 3a–c that all years before the base period did not pass the significance level test, indicating that there was no significant difference between the treatment group and the control group before the implementation of *UAP*. At the same time, the policy effects in years after the base period are all less than 0, indicating that *UAP* has the effect of reducing *EI*. The above analysis shows that the treatment group and the control group meet the conditions of parallel trend hypothesis testing, so the subsequent DID model regression is feasible.

#### 4.2.2. Overall Effect Analysis

Through the parallel trend test mentioned above, this paper conducts the baseline regression of the DID model, as shown in Table 2, where (1) is listed as the regression without control variables, and (2) is listed as the regression result with all control variables.

The *UAP* coefficient in column (1) is significantly less than 0, indicating that *UAP* can significantly reduce *EI*. The first column (2) *UAP* regression coefficient was also significantly less than zero, compared with the first column (1) regression coefficient significantly larger, that of lowering *EI* drops; this may be due to the first column (1) not fully considering the government, industrial structure, the level of economic development and scientific education spending induced errors of regression coefficient is larger. This is similar to the study of Hu and Fan [50]. While they argue that current urban sprawl increases energy consumption, *EI* will break through a critical threshold and gradually decline as cities grow larger. In column (2), government environmental concerns, economic development level, and per capita expenditure on science and education can all reduce *EI*. The reason is that although resource-intensive enterprises and high-energy enterprises in the secondary industry create higher economic benefits, their demand for energy consumption will also increase. Therefore, the more developed the regional industry is, the greater the *EI* will be. The change in industrial structure has a significant impact on *EI*. Luan et al. [51] research results show that the secondary industry increases energy demand, and the *EI* can be effectively inhibited with the increase in the proportion of the tertiary industry. The above analysis shows that *UAP* has a significant effect on reducing *EI*, which verifies the rationality of hypothesis 1.

#### 4.2.3. Heterogeneity Analysis

Under different urban positioning for the study of *UAP* on whether there will be differences in the influence of *EI*, reference Yao et al. [52] research method, in accordance with the “Core–Edge” common structural characteristics, the sample cities are divided into two groups as “Core” and “Edge” city (The “Core” cities include Beijing, Tianjin, Shijiazhuang, Shenyang, Dalian, Changchun, Harbin, Shanghai, Nanjing, Hangzhou, Hefei, Fuzhou, Xiamen, Nanchang, Jinan, Qingdao, Zhengzhou, Luoyang, Wuhan, Changsha, Guangzhou, Shenzhen, Chongqing, and Chengdu, while the other cities are “Edge” cities). Firstly, a heterogeneity analysis model was constructed, and the model construction methods were as follows:

(4) $$EI_{i,t}=\alpha_{2}+\beta_{2}DDD_{i,t}+\overset{8}{\underset{m=5}{\sum }}\theta_{m}control_{n}+\mu_{i}+\lambda_{t}+\varepsilon_{i,t}$$

Secondly, when analyzing the Core city, $DDD_{i,t}=UAP_{i,t}\times Core$. If the city belongs to the core city, Core = 1; otherwise, it is 0. When analyzing the Edge city, $DDD_{i,t}=UAP_{i,t}\times Edge$. If the city belongs to the edge city, Edge = 1; otherwise, it is 0. Finally, model regression is carried out, and the regression results are shown in Table 3.

As can be seen from the regression results in column (1) and column (3), the absolute value of the DDD regression coefficient in core cities is significantly greater than that in “Edge” cities, indicating that *UAP* in core cities are more effective in reducing *EI*. When relevant control variables are added, the absolute value of the DDD regression result coefficient in column (2) decreases, indicating that the effect of *UAP* on reducing the *EI* of core cities decreases after considering the bias of other factors. The regression results in column (4) show that the absolute value of the DDD regression coefficient becomes smaller, indicating that other social, economic, and governmental factors cannot be ignored when *UAP* reduce *EI* in “Edge” cities; these factors will reduce the effect. The absolute value of the DDD regression coefficient in column (4) is still larger than that in column (2), indicating that “Edge” cities reduce *EI* more significantly for urban agglomeration planning. This is because the entry of marginal cities into urban agglomerations promotes the transformation of economic development mode and breaks through the energy consumption threshold [50], making them more prominent in reducing energy consumption in *UAP*. Lv et al. [53] divided Chinese cities into eastern, central, and western cities. The results showed that urbanization in the eastern region had no significant effect on *EI* reduction, while urbanization in the western region was conducive to *EI* reduction. From the perspective of core cities and marginal cities, most core cities are located in the eastern region, which also verifies the weakness of core cities in reducing *EI*. To sum up, *UAP* of “Edge” cities play an important role in reducing *EI*. Therefore, urban agglomeration planning should pay attention to the role of different city positioning in the development process. Due to the expansion of urban agglomeration in recent years, the industrial system of many core cities has become more mature, so the impact on *UAP* to reduce energy consumption is small. Similar results can be obtained from relevant control variables. Although the IS regression coefficients of core cities and “Edge” cities are roughly the same, the per capita expenditure on science and education and the level of economic development of core cities are more significant in reducing *EI*, which may be the reason for the difference in policy effects between the two urban agglomerations.

#### 4.2.4. Analysis of the Mediating Effect

The development of green technology is an important factor in reducing *EI* [49]. The approval of *UAP* promotes technological progress by integrating various resources. Therefore, this paper studies whether the approval of *UAP* achieves the expected purpose of reducing *EI* through green innovation capability (GIC). Furthermore, the following mediation effect model was constructed by referring to the research method of Wang et al. [54]:

(5) $$EI=\alpha_{3}+\beta_{3}UAP_{i,t}+\overset{12}{\underset{m=9}{\sum }}\theta_{m}control_{n}+\varepsilon_{i,t}$$

(6) $$GIC=\alpha_{4}+\beta_{4}UAP_{i,t}+\overset{16}{\underset{m=13}{\sum }}\theta_{m}control_{n}+\varepsilon_{i,t}$$

(7) $$I=\alpha_{5}+\beta_{5}UAP_{i,t}+\gamma GIC_{i,t}+\overset{20}{\underset{m=17}{\sum }}\theta_{m}control_{n}+\varepsilon_{i,t}$$

Among them, *EI* is the logarithmic form of energy consumption intensity, $UAP_{i,t}$ is the dummy variable of Urban agglomeration policy, GIC is the logarithmic form of green innovation ability, $control_{n}$ is the control variable of government attention, industrial structure, economic development level and per capita expenditure on science and education. $\varepsilon_{i,t}$ is the random perturbation term, α is the constant term, β, γ, and $\theta_{m}$ are the regression coefficients of variables, and *i* and *t* represent city and time.

Table 4 reports the path regression results of “*UAP*–GIC–*EI*”, and the regression results pass the significance level test. As can be seen from the results in Column (2), *UAP* significantly improve the ability of green innovation and contribute to the development of urban green innovation technology. As can be seen from the results in column (3), the regression coefficients of *UAP* and GIC are both less than 0, indicating that both can reduce *EI*. The absolute value of *UAP* regression coefficient in column (3) is significantly smaller than that in column (1), which indicates that green innovation ability plays a partially mediating role in the process of reducing *EI* in *UAP*. In terms of the proportion of mediating effect, the proportion of mediating effect is 21.77%, indicating that in the process of *UAP* influencing *EI*, there exists an influence path of “*UAP*–GIC–*EI*”. Hong et al. [55] research on the impact of low-carbon city pilot on *EI* found that low-carbon city pilot policies would reduce *EI* through green technology innovation and technological innovation. Our studies all show that green technology innovation plays a mediating role in reducing *EI*.

### 4.3. Robustness Test

#### 4.3.1. PSM-DID Test

According to the above analysis, the *UAP* has a positive effect on *EI*. Although the treatment group and the control group have satisfied the hypothesis of parallel trend, the *UAP* is not a completely natural event, and there is a certain selection bias. Propensity score matching can find out the control group with very similar characteristics to the treatment group in the research object of the article and then conduct matching analysis, which can effectively reduce the regression bias caused by the selection bias, can better solve the deviation degree of the control variable to the investigated variable, and can well avoid the endogeneity problem [56]. Therefore, this paper adopts two methods of 1:2 nearest neighbor matching and kernel matching to conduct PSM-DID regression. Firstly, GEC, IS, PGDP, and FESE are taken as matching variables in this paper, and then Logit regression is carried out, and then neighbor matching and kernel matching were performed to obtain 2508 and 2509 samples, respectively. Finally, PSM-DID regression was conducted to obtain the regression results, as shown in Table 5. As shown in Table 5, the regression coefficient of *UAP* is significantly negative under the two matching conditions, indicating that *UAP* can effectively reduce urban *EI*. In addition, the regression coefficients of *UAP* are consistent with the symbols in Table 2, and the difference in values is small, which verifies the robustness of the regression results of this paper.

#### 4.3.2. Placebo Test

**Figure 4:**
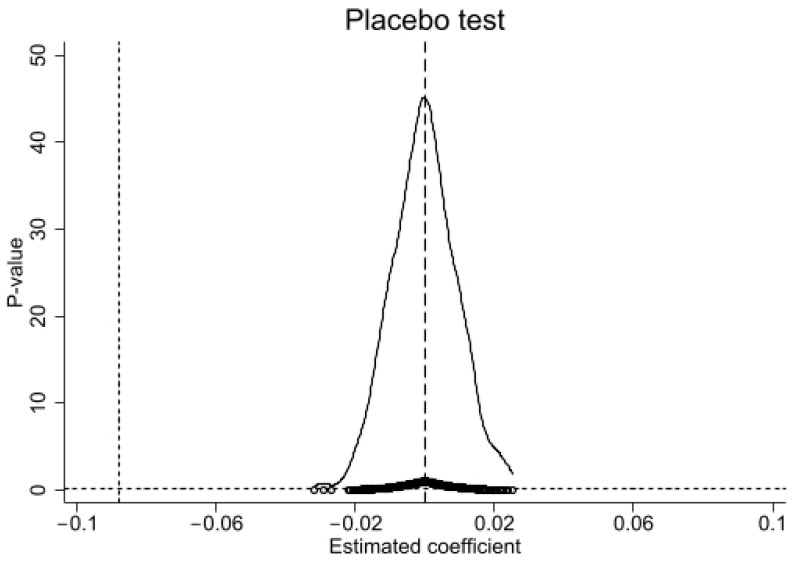
Placebo test results.

The placebo test is usually used to test the robustness of the regression results of the DID model. In order to test whether the *EI* is affected by urban agglomeration planning, the sample cities are randomly sampled 500 times, and the placebo test results are finally obtained, as shown in Figure 4.

As can be seen from Figure 4, the estimation coefficients of *UAP* are roughly normally distributed, and their mean value is close to 0. Meanwhile, the *p*-value of *UAP* estimation coefficient is concentrated near 0, and the *p*-value of most estimates is greater than 0.1. In addition, the regression coefficient of *UAP* was significantly abnormal from the placebo test result, so the study considered the results of this paper to be robust.

#### 4.3.3. Counterfactual Test

In order to eliminate the bias caused by policy-related random factors on the estimation results, this paper conducted a counterfactual test by referring to the research method of Lyu et al. [57] and DID regression on the policy 1–3 years in advance to analyze whether the *UAP* would have a significant impact on *EI*. The regression results are shown in Table 6.

Many scholars have studied the robustness of model regression by advancing policy time. Shi et al. [58] constructed a counterfactual test by advancing the policy time to obtain the robustness of the results. If the regression coefficient after the advance of *UAP* is significantly not 0, it indicates that the reduction of *EI* has been formed before the *UAP*. It can be seen from Table 6 that the regression coefficients of the policies three years in advance failed to pass the significance level test, indicating that *UAP* had an “immediate effect” on reducing *EI*. The regression of DID model met the expected standards, and the regression results were robust.

#### 4.3.4. Instrumental Variable Method

It can be seen from the previous analysis that *UAP* can reduce *EI*, but there are many factors affecting *EI*. In order to solve the endogenous problems caused by omitted variables, this paper adopts the instrumental variable method to solve the problem. The selection of the instrumental variable method should satisfy both correlation and exogeneity assumptions. Therefore, this paper refers to the instrumental variable research method of Yang et al. and Sun et al. [59,60]. From the perspective of geography, “IV(1) = Urban slope × Time” and “IV(2) = Urban altitude × Time” were selected as instrumental variables of *UAP*. The steeper the urban slope and the higher the altitude are, the more unfavorable the construction of urban agglomeration, satisfying the correlation hypothesis. Urban slope and altitude do not directly affect *EI* and generally do not change with time, which satisfies the exogenesis hypothesis. Therefore, it is applicable to select urban slope and urban altitude as instrumental variables. Finally, the dynamic two-step GMM method is used for regression, and the regression results are shown in Table 7.

As can be seen from Table 7, the regression coefficient of *UAP* under the dynamic two-step GMM method is significantly negative compared with Table 2, and the change is small, indicating that *UAP* significantly reduce *EI*. The AR(2) and Hansen statistics of the dynamic two-step GMM regression results failed the significance level test, indicating the externality and effectiveness of the instrumental variables. To sum up, *UAP* do reduce *EI*, which verifies the robustness of the previous research conclusions.

## 5. Conclusions and Suggestions

### 5.1. Conclusions

Taking the policy of urban agglomeration in China as the research object, based on Chinese cities in 2011–2019 panel data, the DID model was used to assess the effect of *UAP* on urban *EI*, analyze the different positioning of the city in *UAP* under the influence of the change of *EI*, and, finally, discuss the policy of urban agglomeration effects on *EI* path. The results show that:

Firstly, on the whole, the approval of *UAP* is conducive to reducing *EI*, which largely reflects the high quality of *UAP* in solving energy consumption problems. The reason is that the development advantage of urban agglomeration is the integration and agglomeration of resources, the agglomeration of advanced production technology and mature industrial structure, and the industrial transfer within urban agglomeration helps to reduce *EI* [61]. This conclusion further corroborates previous similar studies. Urbanization brings economies of scale and creates opportunities to improve energy efficiency [22]. From the perspective of Asian countries, urbanization improves energy efficiency by reducing *EI* [21]. However, contrary to some research conclusions, the *EI* of electricity and coal also increases with the increase in urbanization level [62].

Secondly, the “Core” city and the “Edge” city of urban agglomeration are each made for a different policy to reduce the *EI* effect. The “Core” cities are more mature in terms of economic and social development, gathering a large number of R&D and service institutions and becoming the main base of scientific and technological innovation [63]. Therefore, they are weaker than “Edge” cities in terms of *EI* reduction in *UAP*. The “Edge” cities may have a more significant effect on reducing *EI* after joining urban agglomeration due to their large *EI* base in the early stage. This conclusion confirms the research that the “circle” structure of urban agglomeration reduces *EI*, and this effect will change with the scale density of urban agglomeration [64].

Thirdly, the impact path of *UAP* on *EI* can be summarized as “*UAP*–GIC–*EI*”; that is, *UAP* will reduce *EI* by improving green innovation capability. The ability of green innovation has an obvious inhibiting effect on *EI*, indicating that green technology innovation plays a key role in energy conservation and efficiency improvement. Sun et al. [65] came to a similar conclusion that green innovation helps improve energy efficiency. In addition, Yang’s research conclusion found that green innovation is the mediating mechanism of environmental regulation affecting carbon intensity, and there is a strong mediating effect, which is also very similar to the research conclusion in this paper [66].

### 5.2. Suggestions

Although rapid urban development will increase resource demand, it will also optimize other aspects to offset the resource demand brought by urban development, thus reducing *EI*. Based on the above analysis results, this article puts forward the following suggestions to reduce *EI*.

Firstly, urban agglomeration development planning should be carried out reasonably. The division of urban agglomeration should not only be based on geographical distance but also consider economic distance. As the more closely connected cities have weaker barriers to the flow of economic factors, they will not only continuously improve the level of regional economic development but also enhance the positive effect of urban agglomeration policies on reducing *EI*.

Secondly, the division of different city types should pay attention to their important role in energy consumption. The “Edge” cities should speed up the pace of integration into urban agglomeration and accept radiation from the “Core” cities on industrial and technological innovation.

Thirdly, focus on improving green innovation capacity. Green innovation is related to energy consumption in the process of economic development. Therefore, China should strengthen R&D investment in green technology innovation. Specifically, the government can provide more effective support to relevant green technology innovation enterprises, provide tax incentives and government subsidies for scientific and technological research and development, provide financial support to energy-saving technology enterprises, and further develop and expand the market for green products.

Fourthly, promoting the upgrading of industrial structures is an important measure to curb the surge in *EI*. China’s economic development is restricted by the unbalanced industrial structure, in which the secondary industry accounts for a large proportion, and the high energy consumption enterprises in the secondary industry have a greater demand for energy consumption. Therefore, the proportion of tertiary industry should be increased while the development of high energy consumption industries should be restricted, and the energy demand of high energy consumption industries should be reduced through green technology innovation to further reduce *EI*.

Fifthly, the Chinese government should pay more attention to social and environmental protection and promote the implementation of relevant environmental protection policies, such as carbon emission trading policies and regulations on emission limits for high-energy industries. The government’s emphasis on environmental protection will affect the energy consumption of society. When the government attaches more importance to environmental protection, society may reduce the energy demand to some extent and then make the decision of industrial transformation and upgrading, which is also a powerful measure to reduce *EI*.

In this article, the influence of *UAP* on *EI* in China is discussed by using the DID model. This analytical method and logic can also be used to study the energy conservation and environmental protection problems of other emerging economies because these emerging economies (such as India, Brazil, Vietnam, Indonesia, etc.) may face the same economic development and energy and environmental problems as China. The article still has the following deficiencies: the statistical caliber of the whole electricity consumption of society before and after 2016 in the energy intensity index is inconsistent, which has some research bias. However, this statistical index is more accurate than fitting with light data and other methods. The future research direction is the combination of *UAP* and other policies, which are imposed on urban energy consumption and environmental protection, and the influence and action mechanism on *EI* are also more complex.

## Figures and Tables

**Table 1:** Descriptive statistics of variables.

Variable	Observations	Mean	Std. Dev.	Min	Max
EI	2511	−2.882	0.756	−5.297	2.7
UAP	2511	0.249	0.432	0	1
GEC	2511	−5.821	1.364	−25.328	−4.391
IS	2511	46.924	10.663	11.7	89.34
PGDP	2511	10.699	0.553	8.842	12.223
FESE	2511	8.512	0.595	6.557	10.287
GIC	2511	4.280	1.796	0	10.182

**Table 2:** Regression results of DID.

Variable	EI	
	(1)	(2)
UAP	−0.146 ***	−0.088 **
	(0.044)	(0.040)
GEC		−0.004
		(0.009)
IS		0.008 **
		(0.003)
PGDP		−0.709 ***
		(0.115)
FESE		−0.363 ***
		(0.063)
Cons_	−2.850 ***	7.068 ***
	(0.021)	(1.153)
Observations	2511	2511
R-squared	0.040	0.162
Time fixed	Yes	Yes
City fixed	Yes	Yes

Note: *** = significant at 1% level. ** = significant at 5% level. * = significant at 10% level. Robust standard error in parentheses.

**Table 3:** Heterogeneity regression results of “Core”–“Edge” cities.

Variable	EI			
	“Core” Cities		“Edge” Cities	
	(1)	(2)	(3)	(4)
DDD	−0.128 **	−0.055	−0.122 ***	−0.077 *
	(0.050)	(0.043)	(0.044)	(0.040)
GEC		−0.003		−0.004
		(0.009)		(0.009)
IS		0.008 **		0.008 **
		(0.003)		(0.003)
PGDP		−0.736 ***		−0.719 ***
		(0.115)		(0.115)
FESE		−0.368 ***		−0.366 ***
		(0.061)		(0.062)
Cons_	−2.850 ***	7.384 ***	−2.850 ***	7.196 ***
	(0.021)	(1.152)	(0.021)	(1.149)
Observations	2511	2511	2511	2511
R-squared	0.026	0.157	0.035	0.161
Time fixed	Yes	Yes	Yes	Yes
City fixed	Yes	Yes	Yes	Yes

Note: *** = significant at 1% level. ** = significant at 5% level. * = significant at 10% level. Robust standard error in parentheses.

**Table 4:** Regression results of mediating effects.

Variable	EI	GIC	EI
	(1)	(2)	(3)
UAP	−0.072 **	0.668 ***	−0.057 *
	(0.030)	(0.062)	(0.031)
GIC			−0.024 **
			(0.010)
GEC	−0.010	0.055 ***	−0.008
	(0.009)	(0.019)	(0.009)
IS	0.001	−0.033 ***	−0.0002
	(0.001)	(0.002)	(0.001)
PGDP	0.459 ***	2.110 ***	0.509 ***
	(0.024)	(0.049)	(0.031)
FESE	−0.634 ***	0.218 ***	−0.629 ***
	(0.021)	(0.043)	(0.021)
Cons_	−2.467 ***	−18.433 ***	−2.903 ***
	(0.303)	(0.627)	(0.351)
Proportion of mediating effect	21.77%		
R-squared	0.351	0.507	0.352

Note: *** = significant at 1% level. ** = significant at 5% level. * = significant at 10% level. Standard error in parentheses.

**Table 5:** Regression results of PSM-DID test.

Variable	Nearest Neighbor Matching		Kernel Matching	
	EI		EI	
UAP	−0.149 ***	−0.089 **	−0.148 ***	−0.089 **
	(0.044)	(0.040)	(0.044)	(0.040)
GEC		0.003		0.003
		(0.007)		(0.007)
IS		0.008 **		0.008 **
		(0.003)		(0.003)
PGDP		−0.698 ***		−0.700 ***
		(0.115)		(0.114)
FESE		−0.367 ***		−0.367 ***
		(0.061)		(0.061)
Cons_	−2.850 ***	7.037 ***	−2.850 ***	7.061 ***
	(0.021)	(1.158)	(0.021)	(1.155)
Observations	2508	2508	2509	2509
R-squared	0.041	0.163	0.041	0.164
Time fixed	Yes	Yes	Yes	Yes
City fixed	Yes	Yes	Yes	Yes

Note: *** = significant at 1% level. ** = significant at 5% level. * = significant at 10% level. Robust standard error in parentheses.

**Table 6:** Counterfactual test results.

Variable	EI		
	Policy Advance 1 Year	Policy Advance 2 Year	Policy Advance 3 Year
UAP	−0.067	−0.058	−0.044
	(0.043)	(0.043)	(0.043)
GEC	−0.004	−0.004	−0.003
	(0.009)	(0.009)	(0.009)
IS	0.008 **	0.008 **	0.008 **
	(0.003)	(0.003)	(0.003)
PGDP	−0.725 ***	−0.732 ***	−0.738 ***
	(0.115)	(0.115)	(0.115)
FESE	−0.361 ***	−0.362 ***	−0.366 ***
	(0.063)	(0.063)	(0.062)
Cons_	7.208 ***	7.282 ***	7.384 ***
	(1.153)	(1.152)	(1.149)
Observations	2511	2511	2511
R-squared	0.160	0.159	0.157
Time fixed	Yes	Yes	Yes
City fixed	Yes	Yes	Yes

Note: *** = significant at 1% level. ** = significant at 5% level. * = significant at 10% level. Robust standard error in parentheses.

**Table 7:** Regression results of instrumental variable method.

Variable	Dynamic Two-Step GMM
	EI
UAP	−0.040 ***
	(0.015)
EI_t−1_	0.944 ***
	(0.036)
GEC	−0.007
	(0.004)
IS	0.001
	(0.002)
PGDP	0.061
	(0.057)
FESE	−0.155 **
	(0.078)
Cons_	0.510
	(0.427)
AR(2)	−0.46
	(0.645)
Hansen	107.92
	(0.155)
Time fixed	Yes
City fixed	Yes

Note: *** = significant at 1% level. ** = significant at 5% level. * = significant at 10% level. Robust standard errors in parentheses.

## Data Availability

The data used to support the findings of this study are available from the corresponding author upon request (e-mail: 20201272111047@mail.gufe.edu.cn).
